# SiLaC® with EPSiT®: early outcomes of laser-endoscopic therapy for pilonidal sinus

**DOI:** 10.3389/fsurg.2025.1587467

**Published:** 2025-04-25

**Authors:** Mustapha Ouali

**Affiliations:** Proctolaser Clinic, Sfax, Tunisia

**Keywords:** pilonidal sinus disease, SiLaC®, EPSiT®, laser therapy, minimally invasive surgery, recurrence, patient satisfaction

## Abstract

**Background:**

Pilonidal sinus disease (PSD) commonly affects young adults and often requires repeated interventions with prolonged healing times. We evaluated a combined approach using Sinus Laser Ablation of the Cyst (SiLaC®) and Endoscopic Pilonidal Sinus Treatment (EPSiT®) to investigate postoperative pain, healing time, and recurrence.

**Methods:**

This retrospective, single-center study included 83 patients (aged 15–50) treated from January 2021 to December 2023 at Proctolaser Clinic, Tunisia. Under local, regional, or general anesthesia, patients underwent endoscopic debridement (EPSiT®) followed by diode laser ablation (SiLaC®) of sinus tracts. Data on operative time, Visual Analogue Scale (VAS) pain score, healing time to complete epithelialization, recurrence, and time to return to work were collected. All patients had at least 6 months of follow-up; a subset was followed up to 1 year.

**Results:**

The mean operative time was 25 ± 5.4 min. Mean VAS pain score at 24 h was 1.2 ± 0.6, and 92.3% of patients resumed work or normal activities within 24 h. Mean healing time was 17.3 days, with 95% achieving complete epithelialization by 3 weeks. The overall recurrence rate at 6 months was 3.6% (3/83). Two recurrences (3.3%) occurred in patients with primary PSD, and one recurrence (4.3%) occurred in a patient with recurrent PSD. Minor infections occurred in 1.2% (1/83) of patients and resolved with oral antibiotics. Aesthetic satisfaction was high; 88% rated outcomes “excellent.”

**Conclusions:**

Combining SiLaC® with EPSiT® for pilonidal sinus disease appears safe and effective, featuring minimal pain, rapid return to daily activities, low recurrence, and excellent cosmetic results. A longer-term, multicenter approach is recommended to confirm durability and cost-effectiveness.

## 1 Introduction

Pilonidal sinus disease (PSD) is a chronic inflammatory condition typically arising in the sacrococcygeal region of young adults (15–30 years old) (1, 2). Patients often experience discomfort, local discharge, and recurrent infections, leading to substantial social and economic burdens (3, 4). Traditional management can involve wide excision of sinus tracts with or without flap-based closure; these methods may result in lengthy healing periods and recurrence rates of 20%–30%, in addition to significant postoperative pain (5, 6).

In response, minimally invasive techniques such as laser ablation (SiLaC®) (7–10) and endoscopic debridement (EPSiT®) (11, 12) have emerged. SiLaC® uses a radial laser fiber (commonly 1,470-nm diode) to precisely ablate epithelium in the sinus tract, with minimal injury to surrounding tissue. EPSiT® provides direct endoscopic visualization to thoroughly debride debris, hair, and infected tissue. Although each technique alone can be successful, combining SiLaC® with EPSiT® may permit more complete sinus clearance, potentially reducing recurrences and shortening healing times.

This study evaluates early outcomes and efficacy of the SiLaC® + EPSiT® procedure in 83 patients, focusing on operative time, postoperative pain, healing, recurrence, and patient satisfaction.

Therefore, the primary aim of this study was to evaluate the early clinical outcomes of combining Sinus Laser Ablation of the Cyst (SiLaC®) and Endoscopic Pilonidal Sinus Treatment (EPSiT®) in pilonidal sinus disease, specifically focusing on recurrence rates, healing times, postoperative pain, and patient satisfaction.

## 2 Methods

### 2.1 Study design and setting

We conducted a retrospective single-center study at Proctolaser Clinic, Sfax, Tunisia, from January 2021 to December 2023. The study protocol was reviewed and approved by the National Committee of Medical Ethics of Sfax (Approval No. 27/25) and carried out in accordance with the Helsinki declaration. Written informed consent for surgical treatment and data usage was obtained from each patient. For those under 18, parental or guardian consent was required. All procedures were performed in compliance with the Declaration of Helsinki.

### 2.2 Patient selection

We included patients aged 15–50 years with clinically or endoscopically confirmed PSD (either primary or recurrent). All participants underwent the same combined procedure (SiLaC® + EPSiT®) and agreed to return for regular follow-up visits over at least 6 months. Patients with acute abscesses requiring immediate drainage, major comorbidities affecting wound healing (e.g., severe immunosuppression), or inadequate follow-up data were excluded. A total of 83 patients (60 primary PSD, 23 recurrent PSD) met inclusion criteria.

### 2.3 Surgical procedure

All procedures were conducted in a dedicated operating suite at Proctolaser Clinic, specifically equipped for endoscopic and laser-assisted interventions. Patients were placed in a prone position to clearly expose the sacrococcygeal area. The type of anesthesia—local, regional, or general—was chosen based on patient preference, complexity of the sinus, and anesthesiologist's recommendation.

**Figure 1:**
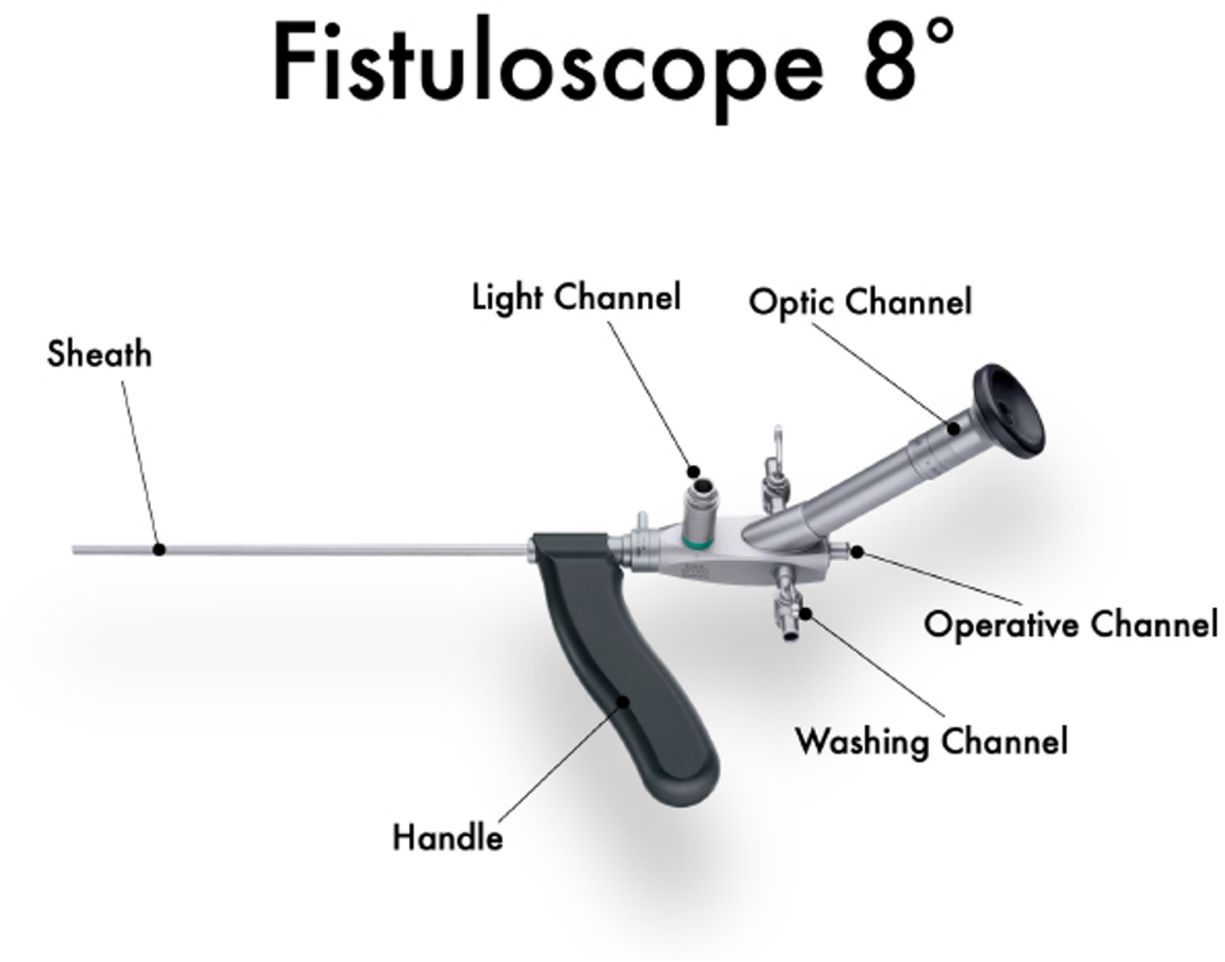
Karl Storz fistuloscope for EPSiT®.

**Figure 2:**
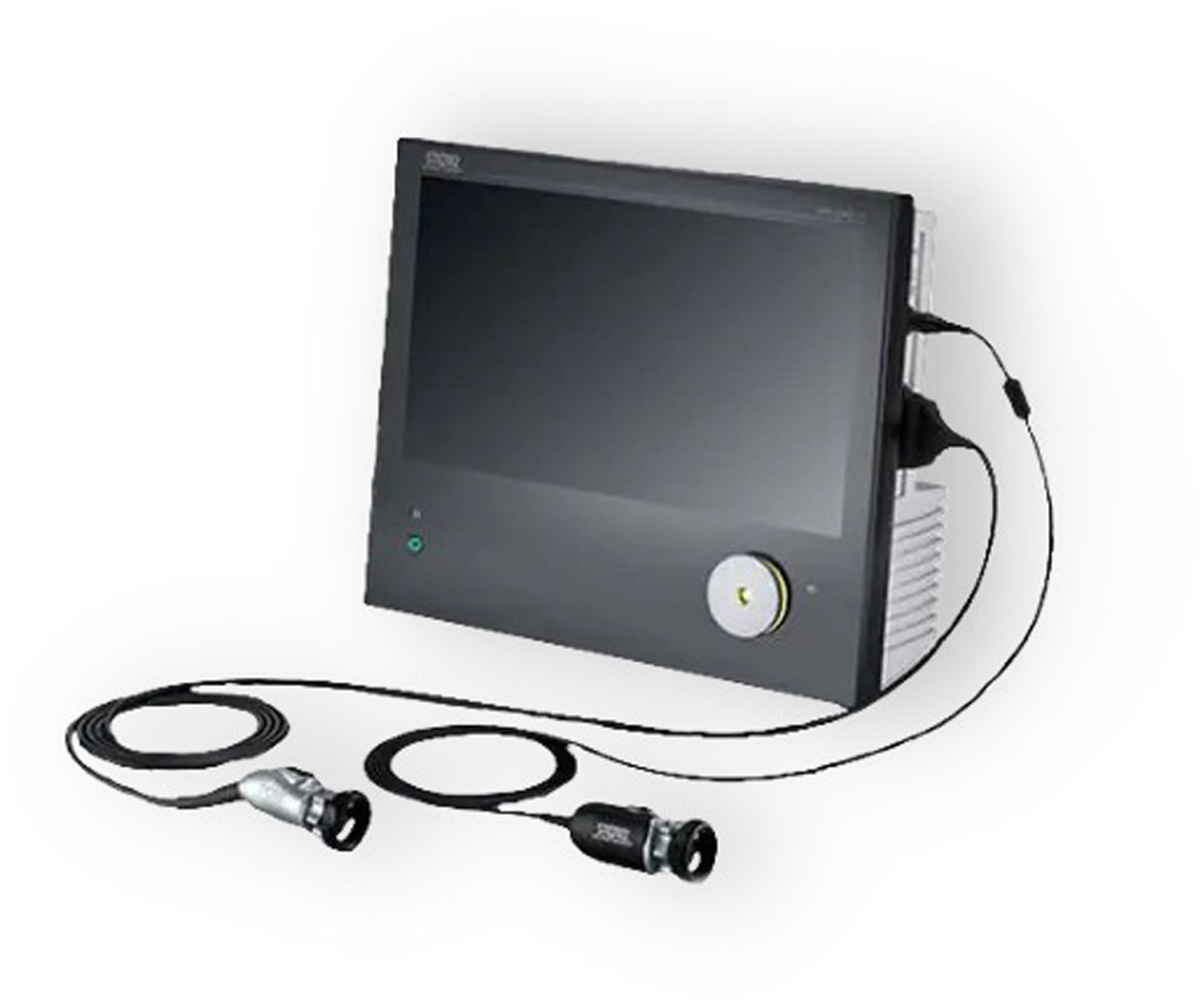
High-definition camera and monitor system.

The intervention commenced by gently dilating the external sinus openings, allowing insertion of a specialized Karl Storz fistuloscope (Figure 1) equipped with a high-definition digital camera system and monitor (Figure 2). This advanced fistuloscope, measuring 3.3 × 4.7 mm in diameter and 18 cm in length with an 8-degree viewing angle, facilitated insertion through small entry points and provided superior visual clarity for the surgeon. Continuous parallel water delivery and suction capabilities ensured a clear operative field by effectively removing hair, debris, and necrotic materials from the sinus tracts. The device's transparent optics and angled eyepiece design enabled detailed inspection and precise manipulation of instruments during surgery, and it complied with rigorous sterilization standards through autoclaving procedures.

**Figure 3:**
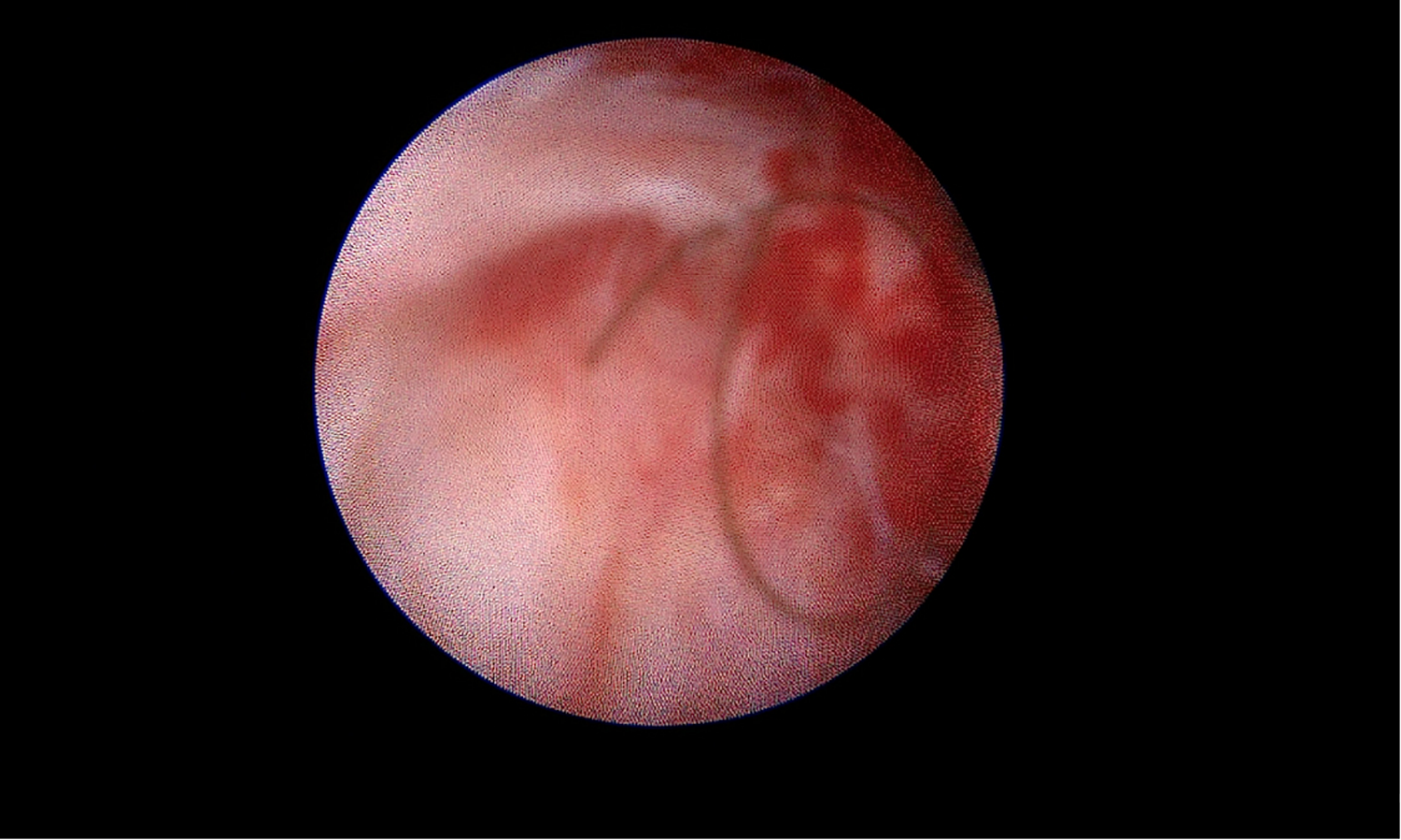
Remaining hair in the fistulous tract after debridement.

**Figure 4:**
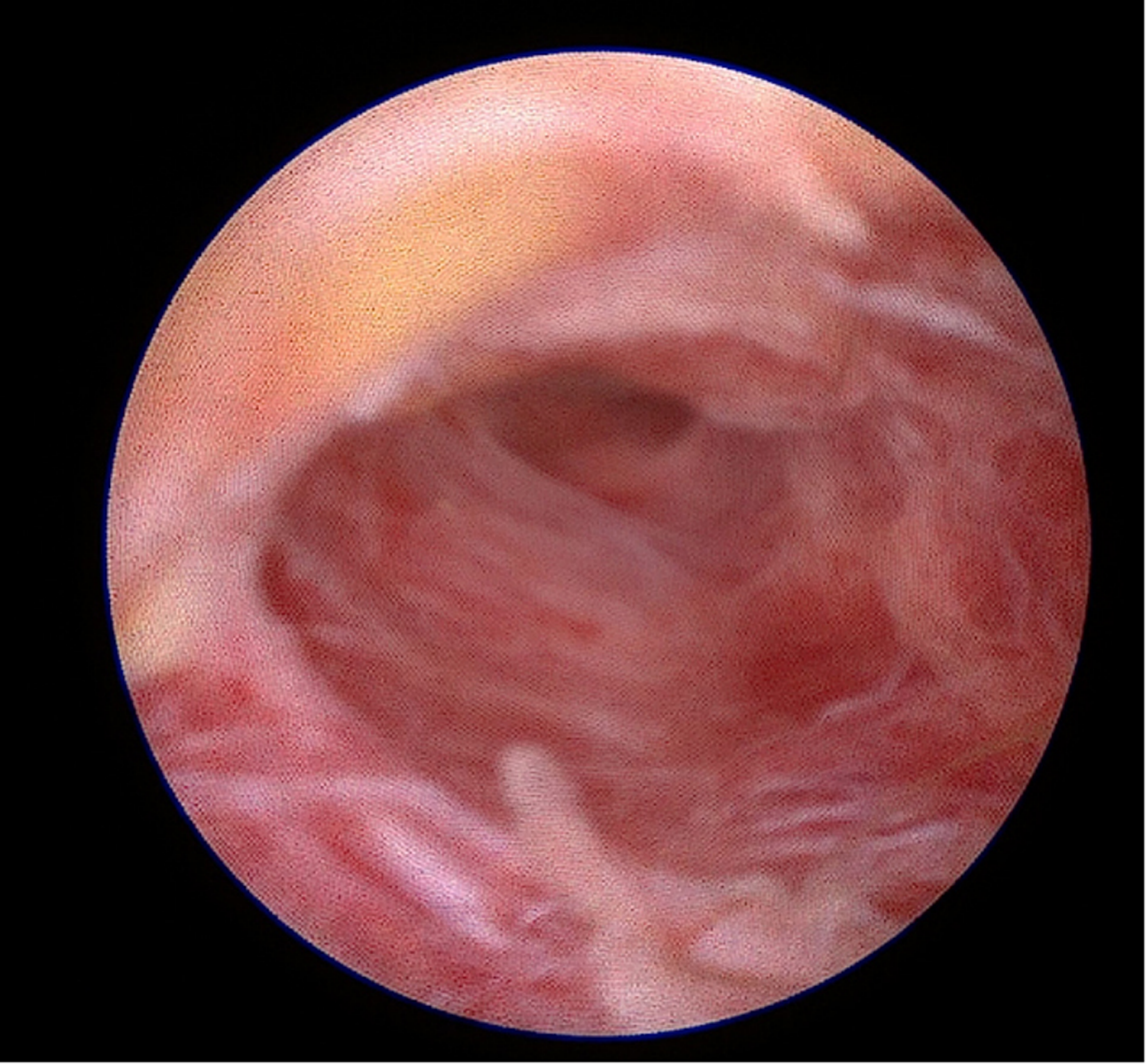
Dedicated vacuum extraction preserving a clear view.

**Figure 5:**
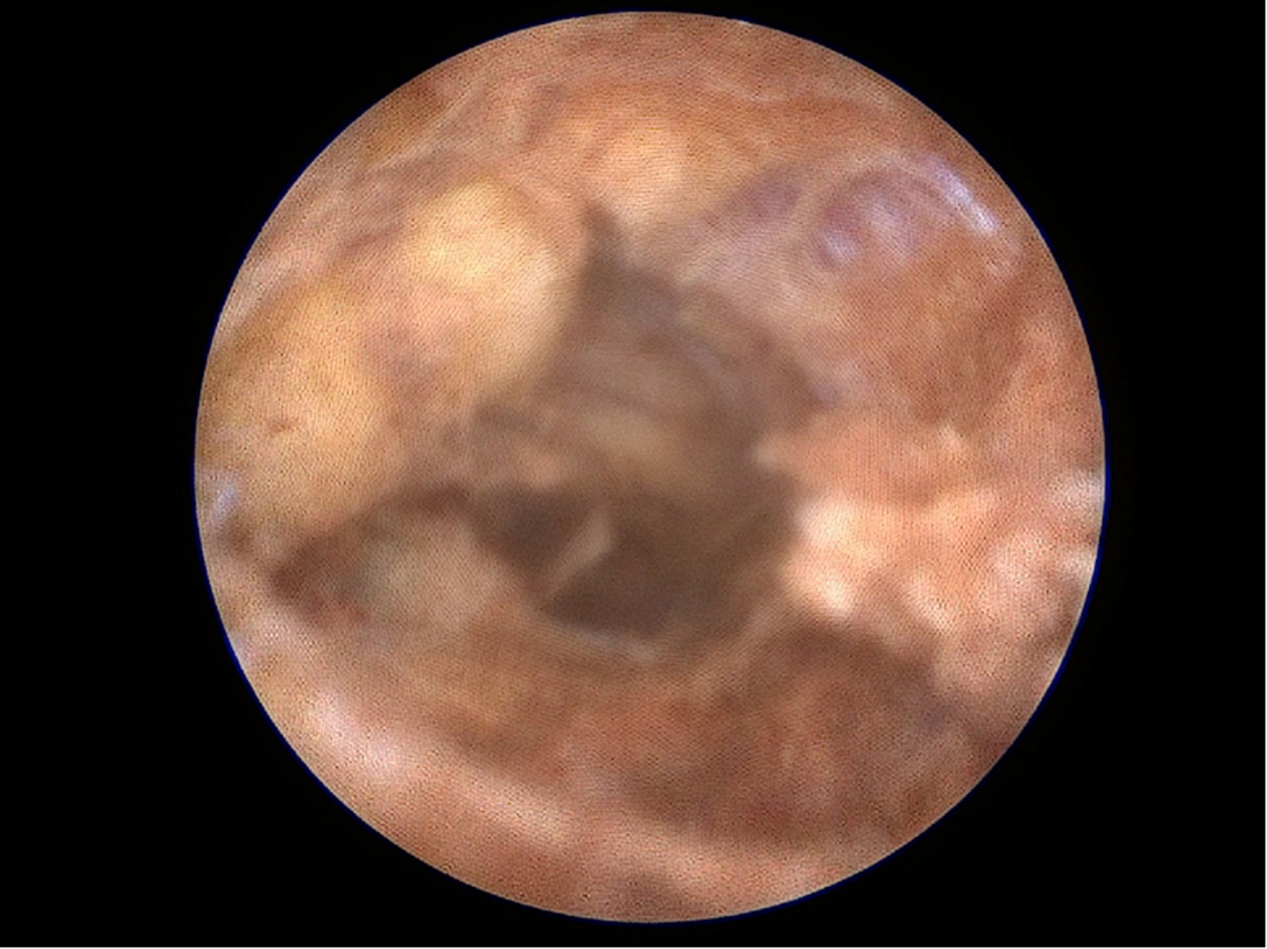
Uniform color change indicating complete laser ablation.

Under direct endoscopic visualization, a thorough inspection was performed to identify all sinus openings and extensions. Fine endoscopic instruments and specialized brushes were employed to meticulously debride and cleanse the sinus tracts, aided by continuous saline irrigation to maintain optimal visibility and remove residual hair and debris (Figure 3). Additionally, a dedicated vacuum system integrated into the endoscope effectively extracted obstructive materials, thereby preserving a consistently clear view throughout the procedure (Figure 4). This meticulous approach adheres strictly to the Endoscopic Pilonidal Sinus Treatment (EPSiT®) principles, which integrate diagnostic examination with therapeutic intervention for comprehensive and effective sinus tract management (13). Endoscopic visualization also allowed real-time monitoring of laser effectiveness, evident by a uniform color change to yellow-brown within the sinus tract walls, indicating complete and homogeneous laser application without untreated zones that could lead to recurrence (Figure 5).

**Figure 6:**
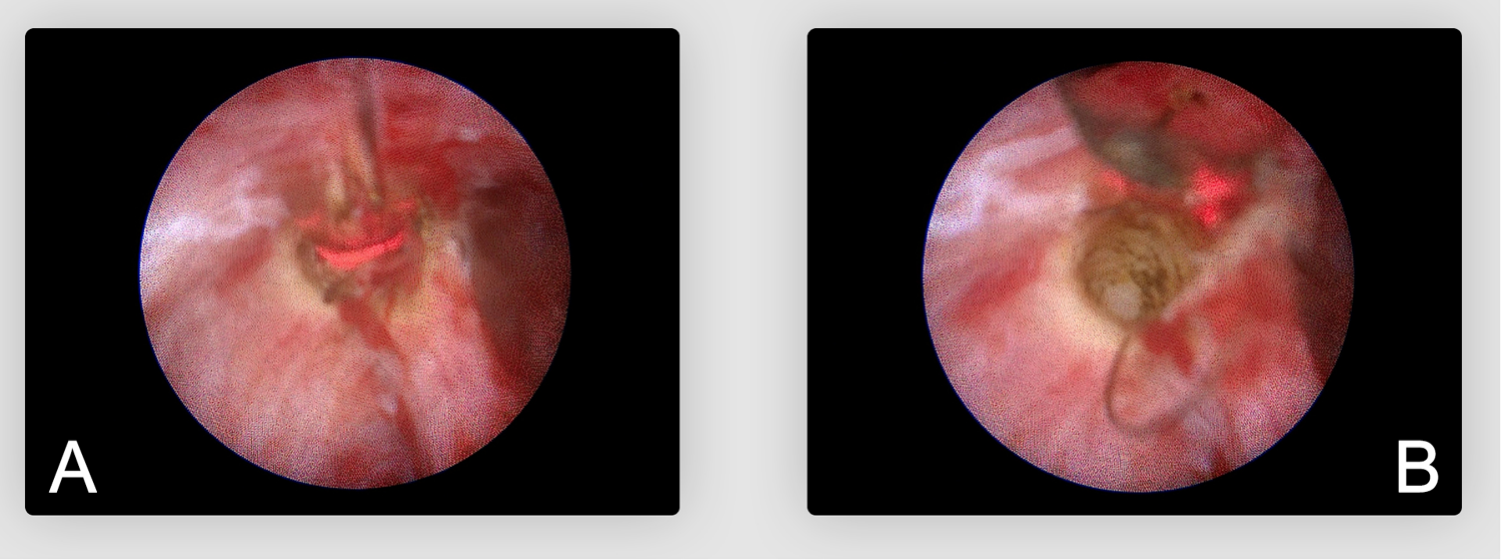
Endoscopic view of uniform laser ablation. **(A)** Radial fiber tip delivering laser energy circumferentially. **(B)** Resulting appearance of the sinus wall after laser ablation.

Following detailed endoscopic debridement, Biolitec's advanced 1,470-nm diode laser system (12), equipped with its proprietary radial fiber tip, was introduced into each sinus tract under direct endoscopic control. The radial fiber tip efficiently delivered energy uniformly in all directions, ensuring comprehensive ablation of sinus tract walls without damage to surrounding healthy tissue (Figure 6). The laser was set to a constant energy output of 10 watts and was methodically withdrawn at a rate of approximately 1 mm per second. Because the irrigation was stopped or minimized, the laser remained in direct contact with the sinus walls, maximizing ablation efficiency. By briefly halting irrigation, we ensured that tissue contact with the fiber tip was not inhibited by fluid, thus preserving optimal radial emission. This meticulous technique precisely removed damaged epithelial tissue, effectively controlled intraoperative bleeding, and promoted tissue shrinkage by specifically targeting water and hemoglobin in cellular structures.

When sinus tracts presented multiple branches or complexity, each segment was individually identified and precisely ablated, ensuring thorough elimination of diseased tissue and reducing recurrence risk. The radial emission pattern and flexible characteristics of the Biolitec® fiber allowed smooth navigation and effective treatment of irregular sinus pathways.

**Figure 7:**
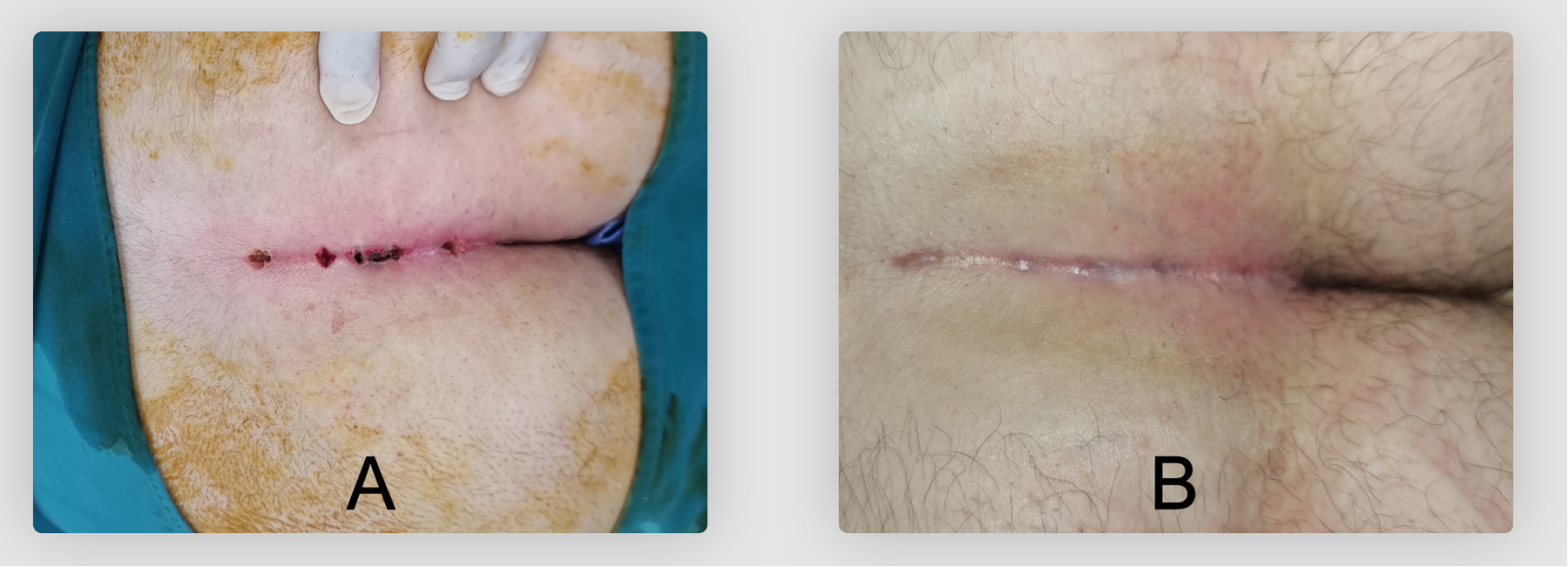
External operative wound appearance after SiLaC® + EPSiT®. **(A)** Immediate postoperative aspect. **(B)** Scar at 60 days postoperatively.

External sinus openings were deliberately left open post-procedure, allowing healing by secondary intention (Figure 7). Postoperatively, sterile dressings and mild compression were applied to facilitate hemostasis. Patients received clear instructions on wound care to maintain cleanliness and dryness, promoting optimal healing conditions. Operative times from initial endoscope insertion to final dressing application were precisely documented.

### 2.4 Postoperative evaluation and follow-up

Patients were generally discharged the same day or within 24 h. Pain was assessed using a 10-point Visual Analogue Scale (VAS) at 24 h, 1 week, and 2 weeks postoperatively. Analgesic consumption (NSAIDs or acetaminophen) was documented. Healing was confirmed by complete epithelialization and absence of discharge or persistent openings; the average time to achieve this state was recorded.

All 83 patients were followed for at least 6 months; some returned for voluntary check-ups up to 1 year. A recurrence was defined as clinical evidence of sinus tract reformation or persistent drainage confirmed by endoscopic evaluation when needed. Any complications (e.g., infection, hematoma, delayed healing, bleeding) were also documented.

### 2.5 Statistical analysis

All data were recorded in a secure database and analyzed using IBM SPSS Statistics version 25.0 (IBM Corp., Armonk, NY). Continuous variables are reported as mean ± standard deviation (SD) or median (range), depending on normality checks. Categorical variables are expressed as counts and percentages. Comparisons between primary and recurrent pilonidal sinus disease (PSD) used the chi-square or Fisher's exact test for categorical variables and the t-test or Mann–Whitney U test for continuous variables. A *p*-value < 0.05 was considered statistically significant. Recurrence rates were summarized descriptively, and no formal survival analysis was performed.

## 3 Results

### 3.1 Demographics and baseline characteristics

Eighty-three patients met inclusion criteria (mean age 28.4 ± 6.2 years, range 15–50). The male-to-female ratio was approximately 1.5:1. A total of 72.3% (60/83) presented with primary PSD, while 27.7% (23/83) had recurrent disease. Most patients have one to three external sinus openings (mean 1.3, range 1–3). Table 1 summarizes baseline variables.

**Table 1:** Patient demographics and baseline characteristics.

Characteristic	Value
Total number of patients	83
Mean age (years)	28.4 ± 6.2 (range: 15–50)
Gender (M:F ratio)	1.5:1
Primary PSD	60 (72.3%)
Recurrent PSD	23 (27.7%)
Mean number of sinus openings	1.3 (range: 1–3)

PSD, pilonidal sinus disease.

### 3.2 Operative outcomes

The overall mean operative time was 25 ± 5.4 min (range 15–45). Local anesthesia was used in 41% (34/83), regional in 34% (28/83), and general in 25% (21/83). During endoscopic inspection, 9.6% (8/83) had previously undetected branching tracts, which were simultaneously debrided and ablated. No intraoperative complications required conversion or additional surgery. Detailed outcomes are reported in Table 2.

**Table 2:** Operative outcomes for patients undergoing combined SiLaC® + EPSiT®.

Operative parameter	Value
Mean operative time (minutes)	25 ± 5.4 (range: 15–45)
Type of anesthesia	Local: 41% (34/83)
	Regional: 34% (28/83)
	General: 25% (21/83)
Secondary tracts discovered intraoperatively	9.6% (8/83)
Intraoperative complications	0%
Mean VAS pain score at 24 h	1.2 ± 0.6
Return to work (within 24 h)	92.3%
Minor infection (superficial)	1.2% (1/83)
Complete healing time (mean ± SD, days)	17.3 ± 3.8 (range 14–28)
Aesthetic satisfaction (Excellent/Satisfied)	88%/10%

- SiLaC® = Sinus Laser ablation of the Cyst.

- EPSiT® = Endoscopic Pilonidal Sinus Treatment.

### 3.3 Pain and return to activities

Postoperative pain was generally mild. At 24 h, the mean VAS score was 1.2 ± 0.6, with only simple oral analgesics (NSAIDs or acetaminophen) needed in most cases. By the end of the first postoperative week, 85% of patients reported a VAS ≤ 1. Notably, 92.3% returned to normal daily activities or work within 24 h—reflecting the minimally invasive nature of the procedure.

### 3.4 Healing time and complications

Complete epithelialization required an average of 17.3 days (range 14–28), with 95% achieving full closure by 3 weeks. Patients with recurrent PSD took slightly longer to heal (18.9 ± 4.1 days) compared to primary PSD (16.5 ± 3.2 days; *p* = 0.03). Only one case (1.2%) experienced a superficial infection; it resolved with oral antibiotics. No hematomas, delayed hemorrhage, or reoperations for wound complications were reported.

### 3.5 Recurrence

Over a minimum follow-up of 6 months, the overall recurrence rate was 3.6% (3/83). Two recurrences (3.3%) occurred in patients with primary PSD, and one recurrence (4.3%) occurred in a patient with recurrent PSD. All cases were successfully managed with repeat laser ablation.

Additional outcomes comparing primary and recurrent PSD are detailed in Table 3. Healing times were significantly shorter in primary PSD (16.5 ± 3.2 days) than in recurrent PSD (18.9 ± 4.1 days, *p* = 0.03). Both groups demonstrated a similar, rapid return to work (generally within 24 h), with minor delays for patients engaged in manual labor. Cosmetic results were highly satisfactory for each group, with at least 95% of patients reporting excellent or satisfactory outcomes. These findings underscore the favorable postoperative profile of the combined SiLaC® and EPSiT® approach for both primary and recurrent PSD.

**Table 3:** Comparison of primary vs. recurrent PSD.

Outcome parameter	Primary PSD (*n* = 60)	Recurrent PSD (*n* = 23)	*p*-value
Mean healing time (days)	16.5 ± 3.2	18.9 ± 4.1	0.03
Return to work within 24 h	93%	91%	0.72
Recurrence rate	3.3% (2/60)	4.3% (1/23)	0.08
Patient satisfaction (excellent/satisfactory)	96.7%	95.6%	0.65

PSD, pilonidal sinus sisease.

### 3.6 Aesthetic outcomes

Most patients (88%) rated their cosmetic result as “excellent,” and an additional 10% were “satisfied.” These ratings were consistent with the clinical examination, which showed minimal scarring and a favorable postoperative appearance. The high aesthetic satisfaction aligns with the fact that only small incisions or openings are needed for EPSiT® and SiLaC®.

## 4 Discussion

Our findings suggest that combining SiLaC® with EPSiT® is a safe and efficient treatment modality for pilonidal sinus disease, offering low recurrence, mild postoperative pain, and swift recovery. The 3.6% overall recurrence rate (exclusively in previously recurrent cases) compares favorably with many flap-based procedures, which can have recurrence rates of 5%–10% or higher (7, 14, 15). Thorough debridement under endoscopic guidance appears instrumental in identifying hidden tracts, while laser ablation precisely treats epithelialized tissue and reduces collateral damage.

### 4.1 Comparison with other techniques

Traditional wide excision often entails longer healing (4–6 weeks) and greater postoperative pain (5, 6). Although flap methods (Karydakis, Bascom) can lower recurrence, they still risk flap necrosis or other complications (14–16). Endoscopic-only (EPSiT®) or laser-only (SiLaC®) approaches have each shown promise, but combining them addresses potential blind spots in endoscopic debridement or incomplete ablation where sinus anatomy is more complex (9–12).

SiLaC® alone has demonstrated good outcomes in terms of low recurrence and faster healing compared to wide excision, but it has limitations. SiLaC® only targets the epithelial lining of the sinus tract without providing direct visualization of secondary or branching tracts. Therefore, incomplete ablation of hidden tracts can lead to recurrence. Lohsiriwat et al. reported a recurrence rate of approximately 5% with SiLaC® alone, which is higher than the 3.6% observed in our study using the combined SiLaC® + EPSiT® approach (10). This suggests that although SiLaC® effectively treats the epithelialized tract, it may not sufficiently address complex or branching sinus anatomy.

In addition, a recent systematic review by Romic et al. (17), which included multiple studies on laser therapy for pilonidal sinus, reinforced that laser ablation yields high short-term success rates and minimal invasiveness while highlighting the need for larger, well-designed trials to confirm long-term outcomes. Our present results with combined laser-endoscopic therapy align with these conclusions and build on them by demonstrating how thorough endoscopic debridement may complement laser ablation to address complex or branching tracts.

EPSiT® alone provides direct visualization, which allows for precise debridement of debris and hair. However, endoscopic debridement alone may leave epithelial remnants that could result in recurrence. Meinero et al. reported a recurrence rate of 6.7% with EPSiT® alone (13). Our data suggest that combining EPSiT® with SiLaC® capitalizes on the strengths of both techniques: EPSiT® ensures thorough debridement of complex tracts, while SiLaC® provides circumferential ablation to eliminate epithelial cells and reduce the risk of recurrence.

A recent comparative study by Ersavas et al. evaluated 73 patients treated with either SiLaC® or EPSiT® and found no significant difference in operative time (32.3 ± 14.8 min for EPSiT® vs. 31.0 ± 14.8 min for SiLaC®; *p* = 0.757), total wound healing time (23.6 ± 14.7 days for EPSiT® vs. 25.2 ± 14.5 days for SiLaC®; *p* = 0.385), or return to daily activities (3.4 ± 0.9 days for EPSiT® vs. 3.6 ± 1.2 days for SiLaC®; *p* = 0.679) (18). However, the need for analgesia was significantly shorter in the EPSiT® group (1.3 ± 0.5 days vs. 1.9 ± 1.1 days for SiLaC®; *p* = 0.005). The recurrence rate was 11.1% in the EPSiT® group and 8.1% in the SiLaC® group (*p* = 0.711), suggesting similar long-term outcomes.

While Ersavas et al.'s study demonstrated comparable success rates, the higher analgesic requirement in SiLaC® suggests that the direct visualization offered by EPSiT® facilitates more effective debridement and potentially faster recovery (18). Our combined approach of SiLaC® + EPSiT® achieved a lower recurrence rate (3.6%), indicating that the synergy of complete endoscopic debridement and circumferential laser ablation may produce superior long-term results.

The 3.6% recurrence rate observed in this study is lower than reported rates for either SiLaC® or EPSiT® alone, indicating that the combination offers a more comprehensive and definitive treatment. This supports the rationale that EPSiT® and SiLaC® are complementary rather than redundant. The combined approach addresses both the structural and epithelial components of the sinus tract, which may explain the enhanced outcomes in terms of lower recurrence, faster healing, and greater patient satisfaction.

A multicenter study comparing SiLaC® alone, EPSiT® alone, and the combined SiLaC® + EPSiT® technique would help confirm these findings and establish clearer guidelines for the optimal surgical approach.

### 4.2 Pain, healing, and return to work

Our patients reported low VAS scores, confirming that targeting disease-specific tracts rather than excising large tissue areas leads to less pain. Over 90% resumed regular activity within 24 h, reflecting minimal disruption to daily life and significant socioeconomic advantages. Healing was substantially faster (mean 17 days) than reported for many traditional excisional techniques, potentially related to the limited tissue trauma of laser-based methods (9).

### 4.3 Study limitations and future directions

As a retrospective, single-center study, our results may not be fully generalizable. The sample size of 83 patients, while sufficient for demonstrating early feasibility, remains modest, particularly for subgroup analyses (e.g., primary vs. recurrent PSD). Longer follow-up is also needed to confirm durable recurrence rates beyond 6 or 12 months. Future multicenter or randomized trials should compare combined SiLaC® + EPSiT® directly with established flap or excisional procedures, ideally including cost-effectiveness analyses to weigh equipment costs against lower requirements for wound care and a quicker return to work.

### 4.4 Cost and health-economic implications

From an economic perspective, laser-based procedures such as SiLaC® often involve higher upfront equipment and consumable costs compared to traditional wide excision. However, these initial expenses may be offset by potential long-term savings, including shorter operative times, fewer postoperative visits, and faster patient return to normal activities. Reduced wound care requirements and minimal sick leave can help lower overall costs for both healthcare systems and employers. Nonetheless, rigorous cost-effectiveness evaluations, preferably in prospective, multicenter settings, are needed to fully assess the financial impact and feasibility of adopting laser-endoscopic methods in diverse clinical environments.

## 5 Conclusions

In this cohort of 83 patients, the combination of SiLaC® (laser ablation) and EPSiT® (endoscopic debridement) for pilonidal sinus disease was associated with:

• A low overall recurrence rate (3.6%)
• Mild postoperative pain (mean VAS ∼1.2 at 24 h)
• Rapid healing (mean ∼17 days) and quick return to normal activities (>90% within 24 h)
• Excellent patient satisfaction, especially regarding aesthetic outcomes

These favorable early outcomes warrant further research through larger-scale, multi-institutional trials with extended follow-up to validate long-term benefits, cost-effectiveness, and reproducibility in diverse surgical settings.

## Data Availability

The raw data supporting the conclusions of this article will be made available by the authors, without undue reservation.
